# A Strategy for Quality Control of *Vespa magnifica* (Smith) Venom Based on HPLC Fingerprint Analysis and Multi-Component Separation Combined with Quantitative Analysis

**DOI:** 10.3390/molecules24162920

**Published:** 2019-08-12

**Authors:** Si-Tong Zhou, Kai Luan, Lian-Li Ni, Ying Wang, Shi-Meng Yuan, Yi-Hao Che, Zi-Zhong Yang, Cheng-Gui Zhang, Zhi-Bin Yang

**Affiliations:** 1 Yunnan Provincial Key Laboratory of Entomological Biopharmaceutical R&D, the National–Local Joint Engineering Laboratory for Entomoceutics, Dali University, Dali 671000, China; 2 Yunnan Provincial 2011 Collaborative Innovation Center for Entomoceutics, Dali University, Dali 671000, China; 3 Innovation Team of New Preparation for Entomological Biopharmaceutical R&D, Dali University, Dali 671000, China

**Keywords:** *Vespa magnifica* (Smith), wasp venom, HPLC fingerprint, similarity analysis, hierarchical clustering analysis, principal component analysis, identification

## Abstract

As a folk medicine of the Jingpo minority in Yunnan province, the venom of *Vespa magnifica* has been commonly used for the treatment of rheumatoid arthritis. Quality standardization of the wasp venom is a necessary step for its pharmaceutical research and development. To control the quality of the wasp venom, a method based on high-performance liquid chromatography (HPLC) was developed for chemical fingerprint analysis. In the chromatographic fingerprinting, chemometrics procedures, including similarity analysis (SA), hierarchical clustering analysis (HCA), and principal component analysis (PCA), were applied to classify 134 batches (S1–S134) of wasp venom from different origins. The HPLC fingerprint method displayed good precision (Relative standard deviation, RSD < 0.27%), stability (in 16 h, RSD < 0.34%), and repeatability (RSD < 1.00%). Simultaneously, four compounds (VMS1, VMS2, VMS3, and VMS4) in the wasp venom were purified and identified. VMS1 was 5-hydroxytryptamine, and the other compounds were three peptides that were sequenced as follows: Gly–Arg–Pro–Hyp–Gly–Phe–Ser–Pro–Phe–Arg–Ile–Asp–NH_2_ (VMS2), Ile–Asn–Leu–Lys–Ala–Ile–Ala–Ala–Leu–Ala–Lys–Lys–Leu–Leu–NH_2_ (VMS3), and Phe–Leu–Pro–Ile–Ile–Gly–Lys–Leu–Leu–Ser–Gly–Leu–Leu–NH_2_ (VMS4). The quantifications for these components were 110.2 mg/g, 26.9 mg/g, 216.3 mg/g, and 58.0 mg/g, respectively. The results of this work indicated that the combination of the chemical fingerprint and quantitative analysis offers a reasonable way to evaluate the quality of wasp venom.

## 1. Introduction

Animal venoms are a particular natural source for the discovery of new drugs, and venom therapy is practiced throughout the world. The venoms of toxic animals such as snakes, spiders, scorpions, and wasps have been widely used in traditional Chinese medicines (TCM) and Ayurvedic medicine for thousands of years [1]. In the past few decades, the venom of wasps has attracted considerable interest all around the world. It has been reported that wasp venoms contain a number of pharmacologically active biomolecules, including amines, small peptides, and even enzymes, allergens, and toxins of high molecular mass [2,3]. Wasp (*Vespa magnifica*, Smith) venom is most widely used to treat rheumatoid arthritis in the medicinal practice of the Jingpo, a Chinese national minority. Many published reports have shown that many active compounds with antimicrobial, hemolytic, and anticoagulant activities can be isolated from wasp venom [4,5,6]. An and Yang et al. independently identified two major allergens and purified a protein with phospholipase-like activity from wasp venom [7,8]. The functional activities of venom are now being studied with the aim of drug discovery. However, few reports have focused on the quality control of wasp venom. Considering its widespread use, there is an urgent need to establish an effective method for standardization of wasp venom in order to carry out more and deeper research.

In recent research, chromatographic fingerprint analysis has been recognized as an innovative, rapid, comprehensive, and characteristic method for the identification and qualification of medicines in developed countries [9]. Where the sources of a crude drug have different ecological habitats, harvest seasons, and climates, so the chemical constituents and their amounts can be different, and wasp venom is similar. The dissimilarity of certain peak positions in the chromatogram from this study showed the distinctions between the test samples. According to the presence or absence of peaks, the chromatographic fingerprint method can be used to distinguish authentic materials from substitutes and adulterants. It is one of the most intuitive methods with which to identify traditional Chinese medicines with complex elements [10]. In the present study, we aimed to establish a chromatographic fingerprint method and use similarity analysis (SA), hierarchical clustering analysis (HCA), and principal component analysis (PCA) to evaluate the quality and authenticity of 134 batches of wasp venom from Yunnan, China. The major components were separated and their structural characterizations and quantitative analyses are reported. It was demonstrated that this research provides a scientific method for the quality control of wasp venom.

## 2. Results and Discussion

### 2.1. Fingerprint Analysis

#### 2.1.1. Methodology Validation

**Figure 1:**
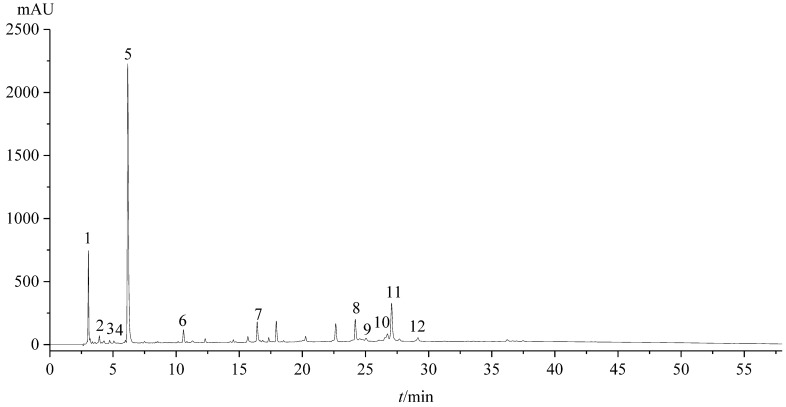
The high-performance liquid chromatography (HPLC) fingerprint chromatogram of wasp venom. Peaks designated: 5 (VMS1); 7 (VMS2); 8 (VMS3); 12 (VMS4). These separations are described further in the Quantified Analysis section.

The production areas and collecting times of wasp venom are different (Table S1). After comparing the chromatograms of the sample solutions several times in detail, we chose 12 common chromatographic peaks from the 134 batches (S1–S134) of wasp venom. Because Peak 5 displayed a better resolution in the middle of the separation gradient and was well separated from adjacent peaks, it was chosen as the reference peak (Figure 1). As shown in Table S2, the precision was determined by analyzing six replicates of a sample solution within the same day to investigate the consistency of the relative retention times of the chromatographic peaks. The relative standard deviation (RSD) values for the relative retention times of 12 chromatographic peaks were calculated to be < 0.27%. The stability was examined by measuring one sample at different time points (0, 2, 4, 10, 12, and 16 h) at room temperature (25 °C ± 3 °C). The RSD values for the stability tests were < 0.34%. The repeatability of method was analyzed by employing six independent sample solutions, and the RSD value was less than 1.00%. All these results indicated that the developed method was precise, stable, and sensitive enough to formulate the fingerprint of wasp venom.

#### 2.1.2. Establishing the Fingerprint

Fingerprint similarity evaluation technology was used to calculate the similarity of 134 batch samples with the 12 common chromatographic peaks. The resulting similarity values were from 0.778 to 0.996 (Table S3). It was indicated that the samples had certain differences because of their different ecological habitats and harvest times, or that adulterants were exist in samples. Not all data were representative, and a fingerprint could not be established directly. In order to establish a credible standard fingerprint, we further analyzed the data with statistical method.

##### Principal Component Analysis

PCA assay was selected for quantitative assessment of the 134 batches of wasp venom, and peak areas of the 12 common peaks obtained by the above method were used to determine the discrimination capacities of the common constituents. PCA converts raw data into new orthogonal variables (principal components, PC), which are combinations of the raw variables. A matrix was applied for the PCA using SPSS software to generate new low-dimensional variables to replace the original high-dimensional variables.

The principle used to extract principal components was that the eigenvalues were greater than 1 or the cumulative variability was higher than 85%. The results showed that the eigenvalues of the first three principal components were all greater than 1: PC1 was 3.722, PC2 was 2.525, and PC3 was 1.690. Thus, the three principal components could be assorted for the samples. The variability of PC1, PC2, and PC3 reached 31%, 21%, and 14%, respectively. The first six principal components contained the most information of all the variables, accounting for 87.047% of the total variability, which indicated that the model can accurately assess datasets. The main chemical markers that had a considerable impact on distinguishing different samples were found using the PCA results. Therefore, we chose the first six components as principal components.

**Figure 2:**
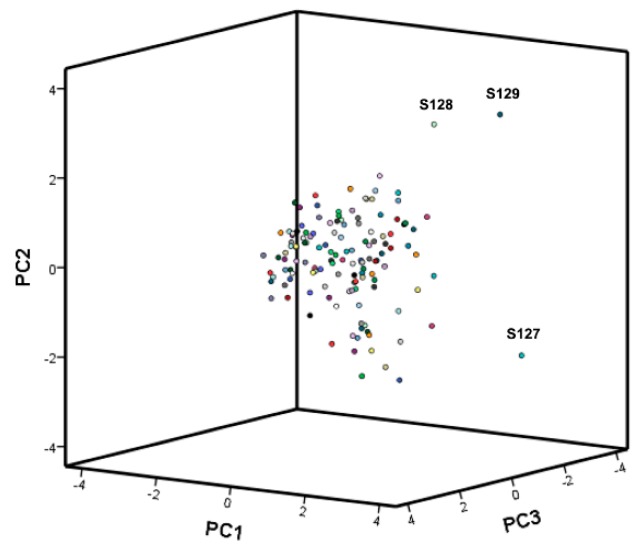
The 3D projection classification map of principal component analysis (PCA) for all the samples.

**Figure 3:**
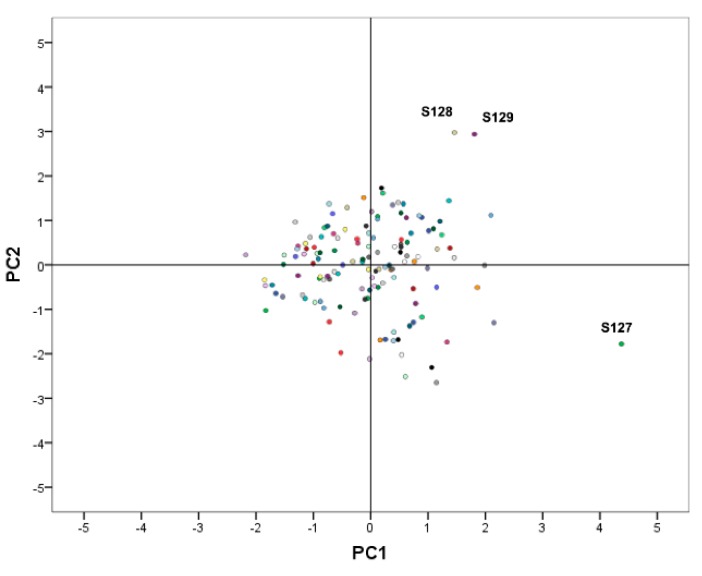
The 2D projection classification map of principal component analysis (PCA) for all the samples.

Once the PCA plan and 3D projection classification map were established, all samples could be obtained by projection (Figure 2 and Figure 3). The intrinsic relationships between the samples were better expressed and classification between samples was realized. As is shown, except for the distances of individual samples S127, S128, and S129, the other distances were relatively concentrated, indicating that there was a significant difference between the samples and a certain degree of correlation.

##### Hierarchical Cluster Analysis

The HCA model could distinguish the samples on the basis of chemical composition to a certain extent, and showed a significant relationship between the chemical composition and the traits. In this study, Ward’s method and the squared Euclidean distance were used to analyze a matrix of 12 × 134. According to the characteristics of the variables, the method classified the degrees of similarity among them. The results showed that 134 samples were divided into four clusters (Figure S1). For the results of the bark, it showed that wasps from different regions could be distinguished by HCA. The venom from Dehong was divided into three parts; most were similar to the venoms of Fuyuan and Wenshan, and another small part was similar to the Baoshan venom. S127 was very different from all other venoms.

##### Establishment of Standard Fingerprint

Referring to the results of the PCA and HCA, 20 batches of the crude wasp venoms with the closest projection distances were used to establish the standard fingerprint. The batch numbers were S7, S13, S16, S20, S24, S34, S39, S40, S41, S42, S46, S49, S58, S61, S72, S81, S85, S99, S104, and S119. The high-performance liquid chromatography (HPLC) fingerprint was established using the Chromatographic Fingerprint of Traditional Chinese Medicine (Version 2012.130723) software. The chromatogram of S46 was used as a reference sample to match the peaks automatically, and the standard template was created by employing the median method to perform multi-point calibration. The fingerprint ultimately had 12 common peaks, with good resolution within 58 min (Figure 1).

#### 2.1.3. Similarity Analysis

The TCM similarity evaluation system was used to assess the similarity values of all the samples from various sources (Table S4). The similarity values of 20 batches of samples were 0.999, 0.998, 0.998, 0.998, 0.997, 0.998, 0.998, 0.997, 0.998, 0.990, 0.999, 0.997, 0.998, 0.997, 0.995, 0.995, 0.997, 0.997, 0.986, and 0.987. As a whole, they were above 0.98, which is in line with the requirements of the Chinese National Pharmacopoeia Commission, and meant that common peaks were in good correlation.

As is shown in Table S4, most of the samples from Dehong, Baoshan, and Wenshan had an acceptable quality level, relatively, and the fingerprint similarity of all samples was greater than 0.900, except for S66, which was 0.890. This indicates that the samples collected from Dehong had similar chemical compositions. In addition, the samples from Dehong produced no noticeable change in the similarity of the sample fingerprints throughout three years at −20 °C without light, in a solid state; thus, the quality of the crude wasp venoms materials was basically stable. The samples from Baise, Fuyuan (except S133) with lower similarities showed weak quality. It was speculated that these samples could be confusion or counterfeit species. 

### 2.2. Quantified Analysis

#### 2.2.1. Identification of Major Components

As Figures S2–S5 show, we ultimately identified VMS1, VMS2, VMS3, and VMS4. The ion at *m*/*z* 177.0768 was the quasi-molecular ion [M + H]^+^ of VMS1 (Figure S6), which was a small molecule. The compound was dissolved in deuterated reagent and analyzed by nuclear magnetic resonance spectrometer.^1^H NMR (400 MHz, MeOD) δ 7.20 (1H, d, *J* = 8.7 Hz, H-7), 7.11 (1H, s, H-2), 6.93 (1H, d, *J* = 2.0 Hz, H-4), 6.71 (1H, dd, *J* = 8.7, 2.3 Hz, H-6), 3.20 (2H, t, *J* = 7.3 Hz, H-11), 3.05 (2H, t, *J* = 7.3 Hz, H-10). ^13^C NMR (101 MHz, MeOD) *δ* 151.52 (C-5), 133.28 (C-8), 128.88 (C-9), 125.04 (C-2), 113.01 (C-3), 112.83 (C-7), 109.39 (C-6), 103.12 (C-4), 41.06 (C-11), 24.61 (C-10). These data demonstrated that the isolated compound was 5-hydroxytryptamine, by comparison with the reported literature [11]. Welsh et al. also reported 5-hydroxytryptamine content in the sting apparatus of *Vespa maculate* [12].

**Figure 4:**
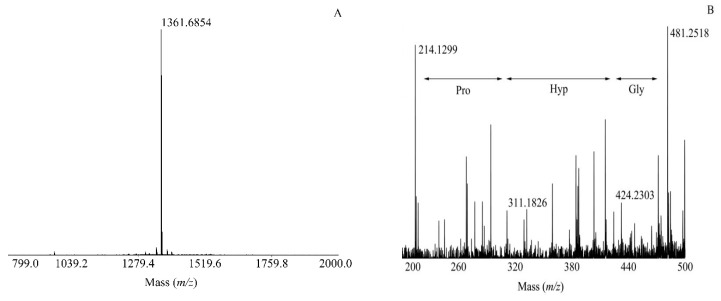
Representative MS and MS/MS analyses of the peptide in this work. (**A**) The matrix-assisted laser desorption/ionization time of flight mass spectrometry (MALDI-TOF-MS) profile of VMS2. The ion at *m*/*z* 1361.6854 was the quasi-molecular ion [M + H]^+^. (**B**) MALDI-TOF-MS/MS characterization of the selected ion (1361.6854) from (A) and the deduced sequence. Lines (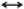) indicate the possible amino acid sequences.

Three peptides were purified from the venom, VMS2, VMS3, and VMS4. They were subjected to Edman sequencing, mass spectrometry (MS/MS), and amino acid determination. These results were defined and verified each other. For example, as shown in Figure 4A, the ion at *m*/*z* 1361.6854 was the quasi-molecular ion [M + H]^+^ of the VMS2. We could obtain a great number of ordered amino acids by Edman degradation. There were twelve amino acid residues in this sequence, H–Gly–Arg–Pro–X–Gly–Phe–Ser–Pro–Phe–Arg–Ile–Asp–OH, although the X could not be identified due to lack of the standard. The accurate structure of the peptide was then validated and determined by MS/MS. The 97.1 Da mass difference between the *m*/*z* 311.1826 and 214.1299 corresponds to a Pro (P) residue, and 57.0 Da mass difference between the *m*/*z* 481.2518 and 424.2303 corresponds to a Gly (G) residue. Accordingly, the *m*/*z* 424.2303 − 311.1826 = 113.0477 constitution of X may be the Hyp/Leu/Ile residue in Figure 4B. However, the retention time of the X was about 6 min in the Edman degradation, which distinguished it from Leu and Ile (18 and 19.5 min). The possibility of Ile or Leu could be precluded. Furthermore, the result of amino acid determination by automatic amino acid analyzer showed that VMS2 contained 8.7% Hyp, which corresponded to one amino acid. This result further proved that the X was Hyp. By combining the above methods, we obtained the full sequence of VMS2, and found that it had been already reported [13,14]. VMS2 was Vespakinin-M, which was the first kinin isolated from insects. It was the first time that Vespakinkin-M has been found in *Vespa magnifica* (Smith) venom. Similarly, VMS3 and VMS4 showed protonated molecular ion peaks at *m*/*z* 1478.9717 and 1382.8684 ([M + H]^+^, monoisotopic, Figures S7 and S8), respectively. As for the identification of Hyp in VMS2, the identification of VMS3 and VMS4 combined Edman degradation, mass spectrometry, and amino acid determination. These results were defined and verified by each other. VMS3, Mastoparan M, was a tetradecapeptide toxin and belongs to the mastoparan homologues of vespid venom. It can induce the release of inflammatory mediators [15]. VMS4 was same as a reported peptide chain from *Vespa mandarinia*, which has significant chemotactic activities for mononuclear leucocytes [16]. It has here been identified in in *Vespa magnifica* (Smith) venom with antimicrobial activity, and named VESCP-M [4]. These compounds are specifically described in Table 1.

#### 2.2.2. Determination of Major Components

The correctness of the quantitative method was confirmed severally by the precision, repeatability, stability, and recovery test. Four samples (VMS1, VMS2, VMS3, and VMS4) were prepared into six different concentrations of solution, and injected into the HPLC in triplicate. The calibration curve (*Y* = a*X* + b) was plotted based on the relative peak areas (*Y*) versus the concentrations of each analyte (*X*). The method of methodology validation was same as the fingerprint analysis, and the recovery was assayed through the standard addition method. The RSDs of the peak areas of the compounds were calculated to evaluate precision, repeatability, stability and recovery, as shown in Table S5. The method was able to completely separate the target chromatographic peaks with good resolution and good repeatability.

The results showed that the four component contents of the different batches of wasp venom were significantly different. The average contents of VMS1, VMS2, VMS3, and VMS4 were 110.2 mg/g, 26.9 mg/g, 216.3 mg/g, 58.0 mg/g, respectively, in the 20 batches of crude venom powder (Table S6). Since the samples were collected from different regions and periods, these differences may be due to diversity in the developmental stages of biology.

## 3. Materials and Methods

### 3.1. Wasps and Crude Venom

The 134 batches (S1–S134) of *Vespa magnifica* (Smith) wasp venom, were collected from different parts of Yunnan Province, China, from July to December of each year from 2012 to 2017. The detailed information of all tested samples is listed in Table S1. The crude venom was obtained using a special device which had light-emitting diodes on the sides and bottom of the unit, and encouraged the wasps to increase their venom release and aggressiveness by flashing lights. It also had an ultrasonic device, which can increase the release of venom. Stimulation was carried out using a voltage of 12 V [17]. The venom was secreted from the tail gland of wasp onto the surface of the glass plate and collected in a special freezer bottle to lyophilize. This special device was independently developed by our team and provided free of charge to farmers. The crude venom was yellow or white and frozen at −20 °C.

### 3.2. Reagents

The HPLC-grade acetonitrile were purchased from Fisher Scientific (The Thermo Fisher Scientific, Inc., Waltham, MA, USA). Trifluoroacetic acid (TFA) was HPLC grade and was obtained from Tedia Company (Tedia Company, Inc., Fairfield, OH, USA). Pure water (18.2 MΩ) for the HPLC analysis was obtained from a water purification system (Merck Millipore, Darmstadt, Hessen, Germany).

### 3.3. Preparation of Sample Solutions

Each of the crude wasp venom powdered samples (7.5 mg) was accurately weighed and dissolved in 1 mL of pure water. The mixture was placed into an ultrasonic bath (32 kHz) for 10 min at 25 °C. After ultrasonic extraction, the extracted solution was centrifuged at 10,000 rpm for 10 min. The supernatant was filtered through a 0.22 μm membrane filter and injected into the HPLC system for analysis.

### 3.4. HPLC Diode Array Detection Analysis Instrumentation

Chromatographic analysis was performed using an Agilent 1260 liquid chromatography system (Agilent Technology, Inc., Santa Clara, CA, USA), which was equipped with a G1311C quat pump, a G1329B autosampler, a G1316A column compartment, and a G1315D diode array detection (DAD) system. The obtained data were analyzed on an Agilent open LAB CDS ChemStation C.01.04. The analysis of fingerprint was carried out at 25 °C on an Agilent ZORBAX 300SB-C18 column (4.6 × 250 mm, 5 μm). The elution gradient of eluents A (acetonitrile with 0.1% TFA) and B (water with 0.1% TFA) was used for the separation of the target analytes. The gradient program was as follows: 0–40 min, 5–77% A; 40–58 min, 77–95% A. Each run was followed by an equilibration time of 5 min. The UV absorbance was monitored at 215 nm, and the solvent flow rate was kept at 1.0 mL/min. All injection volumes of both the samples were 5 μL.

### 3.5. Statistical Analysis

Statistical analysis was applied to demonstrate the variability of the 134 batches of wasp venom samples. On the basis of the HPLC-DAD data, the similarity of these samples was calculated using software named Similarity Evaluation System for Chromatographic Fingerprint of Traditional Chinese Medicine (Version 2012.130723, Chinese Pharmacopoeia Commission, Beijing, China), which was recommended by the National Medical Products Administration (NMPA). Principal component analysis (PCA) was performed on the common chromatographic peaks in the HPLC fingerprints using the IBM SPSS Statistics software (IBM, Version 23, New York, NY, USA). Simultaneously, the hierarchical clustering analysis (HCA) was based on squared Euclidean distance to distinguish samples utilizing IBM SPSS Statistics software (IBM, Version 23, New York, NY, USA).

### 3.6. Purification and Identification

The lyophilized crude venom was dissolved in pure water, filtered and loaded onto a Sepax Bio-C18 column (21.2 × 250 mm, 10 μm). The HPLC was performed on a Waters 2535 system, equipped with a manual injector and two-solvent system: (A) acetonitrile with 0.1% TFA and (B) water with 0.1% TFA [18]. The effluent fractions corresponding to chromatographic peaks were manually collected in tubes, and lyophilized for subsequent testing. Four purified substances were collected in this way, which we designated as VMS1, VMS2, VMS3, and VMS4. Mass measurements of the isolates were performed on a matrix-assisted laser desorption/ionization time of flight mass spectrometry (5800 MALDI-TOF MS, AB Sciex, Framingham, MA, USA). The reflection method with positive ion mode was used to test the molecular mass of the samples. Tandem mass spectrometry (MS/MS) and amino acid determination by automatic amino acid analyzer, together with Edman degradation, were used to analyze the sequences of the purified components.

### 3.7. Determination of Major Components

Venom powder was accurately weighed, transferred into a 2 mL volumetric flask and dissolved with water. The mixture was placed into an ultrasonic bath (32 kHz) for 10 min at 25 °C, and filtered through a 0.22 μm membrane filter as the sample solution prior to HPLC analysis.

The four standard substances were synthesized. The purity and molecular mass of them were checked. Standard solutions were prepared by dissolving 18.20 mg VMS1, 5.50 mg VMS2, 33.00 mg VMS3, and 13.80 mg VMS4, into a 100 mL volumetric flask with acetonitrile aqueous solution to volume, and then filtering.

The four substances in the crude venom were determined by reversed-phase chromatography using the four purified substances as standards. The venom was dissolved in water, and the supernatant was removed after centrifugation. The chromatography was carried out on an Agilent1260 instrument equipped with a diode-array detector. A ZORBAX 300SB-C18 column (4.6 × 250 mm, 5 μm) was eluted at a flow rate of 1 mL/min. The elution was monitored by absorption at 215 nm. A different solvent phase with a non-linear solvent gradient was used for experiments in order to obtain a better separation.

## 4. Conclusions

An HPLC fingerprint method to evaluate the quality of wasp (*Vespa magnifica*) venom was established for the first time. The comprehensive fingerprint analytical techniques used in this study included similarity analysis, PCA, and HCA. The contents of major components provided more references for the quality evaluation of the venom, both quantitatively and qualitatively. The fingerprint contained 12 common peaks, and 4 components were identified. 134 batches of wasp venom successfully set up an effective and reliable quality control method to compare the advantages and disadvantages of materials under different growing conditions. Meanwhile, Mastoparan M had the highest content in the analyzed wasp venoms and might be suitable for quality evaluation of wasp venom. All the results indicate that the approach proposed in this study could be a promising and meaningful reference for the quality control of wasp venom. Wasp venom has great development and commercial potential, and our results can provide a theoretical basis for wasp venom resource utilization, quality evaluation, and development of fine varieties.

## Figures and Tables

**Table 1:** Structure of VMS1, VMS2, VMS3, and VMS4.

Components	Structure	Named	Ref.
VMS1	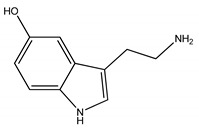	5-Hydroxytryptamine	[11,12]
VMS2	Gly–Arg–Pro–Hyp–Gly–Phe–Ser–Pro–Phe–Arg–Ile–Asp–NH_2_	Vespakinin-M	[13,14]
VMS3	Ile–Asn–Leu–Lys–Ala–Ile–Ala–Ala–Leu–Ala–Lys–Lys–Leu–Leu–NH_2_	Mastoparan M	[15]
VMS4	Phe–Leu–Pro–Ile–Ile–Gly–Lys–Leu–Leu–Ser–Gly–Leu–Leu–NH_2_	Vespid chemotactic peptide M	[4,16]

## Supplementary Materials

The following are available online. Table S1: The distribution of 134 wasp venom samples in Yunnan province of China, Table S2: Method validation for the HPLC fingerprint, Table S3: Preliminary analysis of the similarity of 134 batches of wasp venom, Table S4: The similarity of 134 batches of wasp venom samples, Table S5: Method validation for the quantitative determination of four compounds, Table S6: The contents of VMS1, VMS2, VMS3, and VMS4 in 20 batches of wasp venom samples; Figure S1: Dendrogram of hierarchical cluster analysis (HCA) of 134 batches of wasp venom samples, Figure S2: The liquid chromatogram of VMS1, Figure S3: The liquid chromatogram of VMS2, Figure S4: The liquid chromatogram of VMS3, Figure S5: The liquid chromatogram of VMS4, Figure S6: The MALDI-TOF MS spectra of VMS1, Figure S7: The MALDI-TOF MS spectra of VMS3, Figure S8: The MALDI-TOF MS spectra of VMS4.
