# Supplementary material for: A Strategy for Quality Control of Vespa magnifica (Smith) Venom Based on HPLC Fingerprint Analysis and Multi-Component Separation Combined with Quantitative Analysis
Source: Molecules. 2019 Aug 12;24(16):2920. doi: 10.3390/molecules24162920 (PMC6719070; doi:10.3390/molecules24162920)
Supplement: Supplementary file 1 [file molecules-24-02920-s001.pdf]

Supporting Information for

A Strategy for Quality Control of *Vespa magnifica* (Smith) Venom Based on HPLC Fingerprint Analysis and Multi-Component Separation Combined with Quantitative Analysis

Si-Tong Zhou [1,†], Kai Luan [1,†], Lian-Li Ni [1], Ying Wang [1], Shi-Meng Yuan [1], Yi-Hao Che [1], Zi-Zhong Yang [1,2], Cheng-Gui Zhang[1,2,*] and Zhi-Bin Yang [1,2,3,*]

[1] Yunnan Provincial Key Laboratory of Entomological Biopharmaceutical R&D, the National–Local Joint Engineering Laboratory for Entomoceutics, Dali University, Dali 671000, China

[2] Yunnan Provincial 2011 Collaborative Innovation Center for Entomoceutics, Dali University, Dali 671000, China

[3] Innovation Team of New Preparation for Entomological Biopharmaceutical R&D, Dali University, Dali 671000, China

* Correspondence: chenggui_zcg@163.com (C.-G.Z.); yangzhibin@dali.edu.cn (Z.-B.Y.); Tel.: +86-087-22214251 (C.-G.Z.); +86-087-22214251 (Z.-B.Y.)

† These authors contributed equally to this work.

Contents of Supporting Information

No.	Contents	Pages
1	Table S1. The distribution of 134 wasp venom samples in Yunnan province of China.	3
2	Table S2. Method validation for the HPLC fingerprint.	4
3	Table S3. Preliminary analysis of the similarity of 134 batches wasp venom.	5
4	Table S4. The similarity of 134 batches wasp venom samples.	6
5	Table S5. Method validation for the quantitative determination of four compounds.	7
6	Table S6. The content of VMS1, VMS2, VMS3 and VMS4 of 20 batches wasp venom samples.	8
7	Figure S1. Dendrogram of hierarchical cluster analysis (HCA) of 134 batches wasp venom samples.	9
8	Figure S2. The liquid chromatogram of VMS1.	10
9	Figure S3. The liquid chromatogram of VMS2.	11
10	Figure S4. The liquid chromatogram of VMS3.	12
11	Figure S5. The liquid chromatogram of VMS4.	13
12	Figure S6. The MALDI-TOF MS spectra of VMS1.	14
13	Figure S7. The MALDI-TOF MS spectra of VMS3.	15
14	Figure S8. The MALDI-TOF MS spectra of VMS4.	16

Table S1. The distribution of 134 wasp venom samples in Yunnan province of China.

No.	Sample source	Collection time	No.	Sample source	Collection time	No.	Sample source	Collection time	No.	Sample source	Collection time	No.	Sample source	Collection time
S1	Dehong	2015.11	S28	Dehong	2017.01	S55	Dehong	2017.01	S82	Dehong	2017.01	S109	Baoshan	2012.10
S2	Dehong	2015.11	S29	Dehong	2017.01	S56	Dehong	2017.01	S83	Dehong	2017.01	S110	Baoshan	2012.10
S3	Dehong	2015.11	S30	Dehong	2017.01	S57	Dehong	2017.01	S84	Dehong	2017.01	S111	Baoshan	2012.10
S4	Dehong	2015.11	S31	Dehong	2017.01	S58	Dehong	2017.01	S85	Dehong	2017.01	S112	Baoshan	2012.10
S5	Dehong	2015.11	S32	Dehong	2017.01	S59	Dehong	2017.01	S86	Dehong	2017.01	S113	Baoshan	2012.10
S6	Dehong	2015.11	S33	Dehong	2017.01	S60	Dehong	2017.01	S87	Dehong	2017.01	S114	Baoshan	2012.10
S7	Dehong	2017.01	S34	Dehong	2017.01	S61	Dehong	2017.01	S88	Dehong	2017.01	S115	Baoshan	2012.10
S8	Dehong	2017.01	S35	Dehong	2017.01	S62	Dehong	2017.01	S89	Dehong	2017.01	S116	Baoshan	2012.10
S9	Dehong	2017.01	S36	Dehong	2017.01	S63	Dehong	2017.01	S90	Dehong	2017.01	S117	Baoshan	2012.10
S10	Dehong	2017.01	S37	Dehong	2017.01	S64	Dehong	2017.01	S91	Dehong	2017.01	S118	Wenshan	2013.11
S11	Dehong	2017.01	S38	Dehong	2017.01	S65	Dehong	2017.01	S92	Dehong	2017.01	S119	Wenshan	2013.11
S12	Dehong	2017.01	S39	Dehong	2017.01	S66	Dehong	2017.01	S93	Dehong	2017.01	S120	Wenshan	2013.11
S13	Dehong	2017.01	S40	Dehong	2017.01	S67	Dehong	2017.01	S94	Dehong	2017.01	S121	Wenshan	2013.11
S14	Dehong	2017.01	S41	Dehong	2017.01	S68	Dehong	2017.01	S95	Dehong	2017.01	S122	Wenshan	2013.11
S15	Dehong	2017.01	S42	Dehong	2017.01	S69	Dehong	2017.01	S96	Dehong	2017.01	S123	Wenshan	2013.11
S16	Dehong	2017.01	S43	Dehong	2017.01	S70	Dehong	2017.01	S97	Dehong	2017.01	S124	Wenshan	2013.11
S17	Dehong	2017.01	S44	Dehong	2017.01	S71	Dehong	2017.01	S98	Dehong	2017.01	S125	Wenshan	2013.11
S18	Dehong	2017.01	S45	Dehong	2017.01	S72	Dehong	2017.01	S99	Dehong	2017.01	S126	Wenshan	2013.11
S19	Dehong	2017.01	S46	Dehong	2017.01	S73	Dehong	2017.01	S100	Dehong	2017.01	S127	Wenshan	2013.11
S20	Dehong	2017.01	S47	Dehong	2017.01	S74	Dehong	2017.01	S101	Dehong	2017.01	S128	Wenshan	2013.11
S21	Dehong	2017.01	S48	Dehong	2017.01	S75	Dehong	2017.01	S102	Dehong	2017.01	S129	Baise	2015.10
S22	Dehong	2017.01	S49	Dehong	2017.01	S76	Dehong	2017.01	S103	Dehong	2017.01	S130	Dehong	2014.12
S23	Dehong	2017.01	S50	Dehong	2017.01	S77	Dehong	2017.01	S104	Dehong	2017.01	S131	Fuyuan	2016.12
S24	Dehong	2017.01	S51	Dehong	2017.01	S78	Dehong	2017.01	S105	Dehong	2017.01	S132	Fuyuan	2016.12
S25	Dehong	2017.01	S52	Dehong	2017.01	S79	Dehong	2017.01	S106	Dehong	2012.01	S133	Fuyuan	2016.12
S26	Dehong	2017.01	S53	Dehong	2017.01	S80	Dehong	2017.01	S107	Dehong	2012.01	S134	Fuyuan	2016.12
S27	Dehong	2017.01	S54	Dehong	2017.01	S81	Dehong	2017.01	S108	Baoshan	2012.10			

Table S2. Method validation for the HPLC fingerprint.

**Method validation**	**The retention time of 12 peaks**											
	**No.1**	**No.2**	**No.3**	**No.4**	**No.5**	**No.6**	**No.7**	**No.8**	**No.9**	**No.10**	**No.11**	**No.12**
Precision	0.481	0.506	0.635	0.788	1.000	1.809	2.614	3.842	3.996	4.256	4.307	4.630
	0.482	0.507	0.635	0.785	1.000	1.810	2.618	3.853	4.009	4.271	4.322	4.646
	0.481	0.506	0.635	0.787	1.000	1.807	2.611	3.844	3.999	4.261	4.313	4.636
	0.481	0.506	0.635	0.787	1.000	1.806	2.610	3.847	4.000	4.265	4.315	4.638
	0.484	0.508	0.636	0.785	1.000	1.813	2.623	3.865	4.019	4.282	4.333	4.660
	0.483	0.507	0.634	0.785	1.000	1.807	2.618	3.857	4.012	4.274	4.325	4.650
RSD(%)	0.26	0.16	0.10	0.17	0.00	0.14	0.19	0.23	0.22	0.22	0.22	0.23
Stability	0.481	0.506	0.635	0.788	1.000	1.809	2.614	3.842	3.996	4.256	4.307	4.630
	0.481	0.506	0.635	0.787	1.000	1.807	2.611	3.845	3.999	4.261	4.313	4.636
	0.484	0.508	0.636	0.785	1.000	1.813	2.623	3.865	4.019	4.282	4.333	4.660
	0.484	0.509	0.636	0.785	1.000	1.815	2.626	3.869	4.023	4.287	4.337	4.665
	0.484	0.509	0.636	0.785	1.000	1.815	2.626	3.869	4.023	4.287	4.337	4.665
	0.484	0.508	0.636	0.787	1.000	1.809	2.623	3.863	4.017	4.281	4.332	4.659
RSD(%)	0.32	0.27	0.08	0.17	0.00	0.19	0.24	0.31	0.30	0.32	0.30	0.33
Repeatability	0.476	0.502	0.633	0.796	1.000	1.776	2.570	3.779	3.927	4.180	4.231	4.551
	0.478	0.502	0.632	0.794	1.000	1.771	2.571	3.782	3.933	4.188	4.239	4.559
	0.482	0.506	0.632	0.791	1.000	1.788	2.597	3.823	3.973	4.234	4.286	4.608
	0.485	0.511	0.638	0.789	1.000	1.804	2.610	3.841	3.997	4.256	4.305	4.631
	0.489	0.513	0.639	0.784	1.000	1.806	2.629	3.871	4.025	4.288	4.338	4.667
	0.487	0.513	0.640	0.785	1.000	1.801	2.614	3.856	4.008	4.269	4.319	4.645
RSD(%)	1.06	1.02	0.59	0.61	0.00	0.84	0.92	1.00	1.01	1.04	1.01	1.02

Table S3. Preliminary analysis of the similarity of 134 batches wasp venom.

No.	Similarity	No.	Similarity	No.	Similarity	No.	Similarity	No.	Similarity	No.	Similarity	No.	Similarity	No.	Similarity
S1	0.989	S18	0.984	S35	0.989	S52	0.989	S69	0.993	S86	0.989	S103	0.989	S120	0.942
S2	0.989	S19	0.994	S36	0.991	S53	0.985	S70	0.990	S87	0.988	S104	0.981	S121	0.984
S3	0.993	S20	0.996	S37	0.993	S54	0.990	S71	0.991	S88	0.982	S105	0.985	S122	0.954
S4	0.995	S21	0.993	S38	0.992	S55	0.986	S72	0.990	S89	0.982	S106	0.989	S123	0.987
S5	0.994	S22	0.994	S39	0.994	S56	0.987	S73	0.990	S90	0.985	S107	0.992	S124	0.978
S6	0.994	S23	0.985	S40	0.992	S57	0.992	S74	0.987	S91	0.991	S108	0.984	S125	0.986
S7	0.995	S24	0.993	S41	0.993	S58	0.993	S75	0.981	S92	0.989	S109	0.982	S126	0.980
S8	0.990	S25	0.990	S42	0.987	S59	0.986	S76	0.986	S93	0.987	S110	0.967	S127	0.953
S9	0.994	S26	0.994	S43	0.986	S60	0.994	S77	0.988	S94	0.987	S111	0.989	S128	0.970
S10	0.994	S27	0.985	S44	0.990	S61	0.990	S78	0.986	S95	0.992	S112	0.975	S129	0.812
S11	0.993	S28	0.992	S45	0.984	S62	0.990	S79	0.989	S96	0.983	S113	0.973	S130	0.989
S12	0.990	S29	0.985	S46	0.994	S63	0.987	S80	0.987	S97	0.987	S114	0.979	S131	0.790
S13	0.994	S30	0.988	S47	0.99	S64	0.989	S81	0.987	S98	0.994	S115	0.980	S132	0.866
S14	0.994	S31	0.993	S48	0.983	S65	0.985	S82	0.992	S99	0.990	S116	0.986	S133	0.952
S15	0.989	S32	0.987	S49	0.991	S66	0.901	S83	0.991	S100	0.994	S117	0.985	S134	0.778
S16	0.993	S33	0.993	S50	0.990	S67	0.990	S84	0.985	S101	0.978	S118	0.991		
S17	0.985	S34	0.995	S51	0.994	S68	0.983	S85	0.991	S102	0.988	S119	0.984		

Table S4. The similarity of 134 batches wasp venom samples.

No.	Similarity	No.	Similarity	No.	Similarity	No.	Similarity	No.	Similarity	No.	Similarity	No.	Similarity	No.	Similarity
S1	0.987	S18	0.992	S35	0.990	S52	0.991	S69	0.993	S86	0.996	S103	0.995	S120	0.947
S2	0.989	S19	0.999	S36	0.992	S53	0.990	S70	0.996	S87	0.995	S104	0.985	S121	0.986
S3	0.992	S20	0.998	S37	0.992	S54	0.994	S71	0.991	S88	0.983	S105	0.990	S122	0.952
S4	0.995	S21	0.998	S38	0.998	S55	0.986	S72	0.994	S89	0.981	S106	0.997	S123	0.988
S5	0.997	S22	0.998	S39	0.998	S56	0.990	S73	0.994	S90	0.988	S107	0.997	S124	0.981
S6	0.995	S23	0.991	S40	0.997	S57	0.998	S74	0.993	S91	0.997	S108	0.980	S125	0.987
S7	0.998	S24	0.996	S41	0.998	S58	0.998	S75	0.981	S92	0.995	S109	0.979	S126	0.981
S8	0.988	S25	0.994	S42	0.989	S59	0.992	S76	0.989	S93	0.994	S110	0.964	S127	0.953
S9	0.995	S26	0.997	S43	0.990	S60	0.998	S77	0.995	S94	0.992	S111	0.987	S128	0.974
S10	0.997	S27	0.990	S44	0.991	S61	0.996	S78	0.993	S95	0.997	S112	0.972	S129	0.812
S11	0.994	S28	0.997	S45	0.989	S62	0.995	S79	0.995	S96	0.982	S113	0.970	S130	0.987
S12	0.992	S29	0.993	S46	0.998	S63	0.990	S80	0.993	S97	0.993	S114	0.978	S131	0.775
S13	0.998	S30	0.992	S47	0.997	S64	0.996	S81	0.994	S98	0.997	S115	0.975	S132	0.851
S14	0.999	S31	0.997	S48	0.985	S65	0.993	S82	0.997	S99	0.996	S116	0.985	S133	0.949
S15	0.995	S32	0.987	S49	0.997	S66	0.890	S83	0.996	S100	0.999	S117	0.989	S134	0.765
S16	0.998	S33	0.994	S50	0.996	S67	0.996	S84	0.981	S101	0.977	S118	0.991		
S17	0.991	S34	0.999	S51	0.997	S68	0.990	S85	0.996	S102	0.993	S119	0.986		

Table S5. Method validation for the quantitative determination of four compounds.

**Componds**	**Calibration curve**	**r**	**LOD（μg）**	**LOQ（μg）**	**Linear range（μg）**	**Precision RSD（%)（n = 6）**	**Repeatability RSD（%）（n = 6）**	**Stability RSD（%）（n = 6）**	**Recovery(%)（n = 6）Mean±RSD（%）**
VMS1	*Y* = 4108.6*X* - 410.56	0.9998	0.0336	0.112	0.910 – 4.816	0.98	0.52	0.24	100.8±2.48
VMS2	*Y* = 1285.7*X* + 28.00	0.9984	0.0183	0.055	0.275 – 2.750	1.82	3.15	0.32	99.7±3.44
VMS3	*Y* = 358.5*X* - 35.24	1.0000	0.0990	0.200	1.650 – 9.900	2.73	3.34	0.77	100.7±2.08
VMS4	*Y* = 554.2*X* + 12.83	0.9999	0.2300	0.552	0.690 – 6.900	0.32	2.96	0.28	101.0±2.14

Table S6. The content of VMS1, VMS2, VMS3 and VMS4 of 20 batches wasp venom samples (n = 20).

Componds	Amount（mg/g）	RSD（%）
VMS1	109.1±2.51	2.30
VMS2	26.3±0.81	3.07
VMS3	216.3±1.86	0.86
VMS4	58.0±1.73	2.99

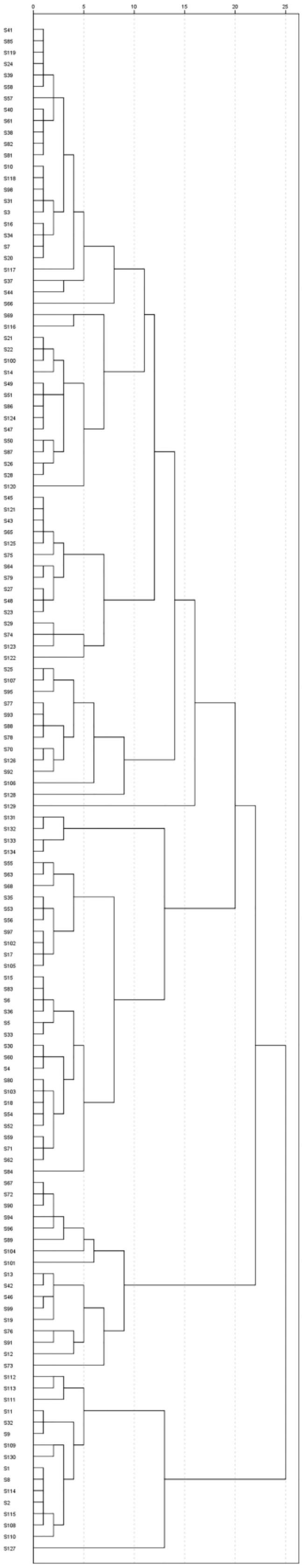

Figure S1. Dendrogram of hierarchical cluster analysis (HCA) of 134 batches of wasp venom samples.

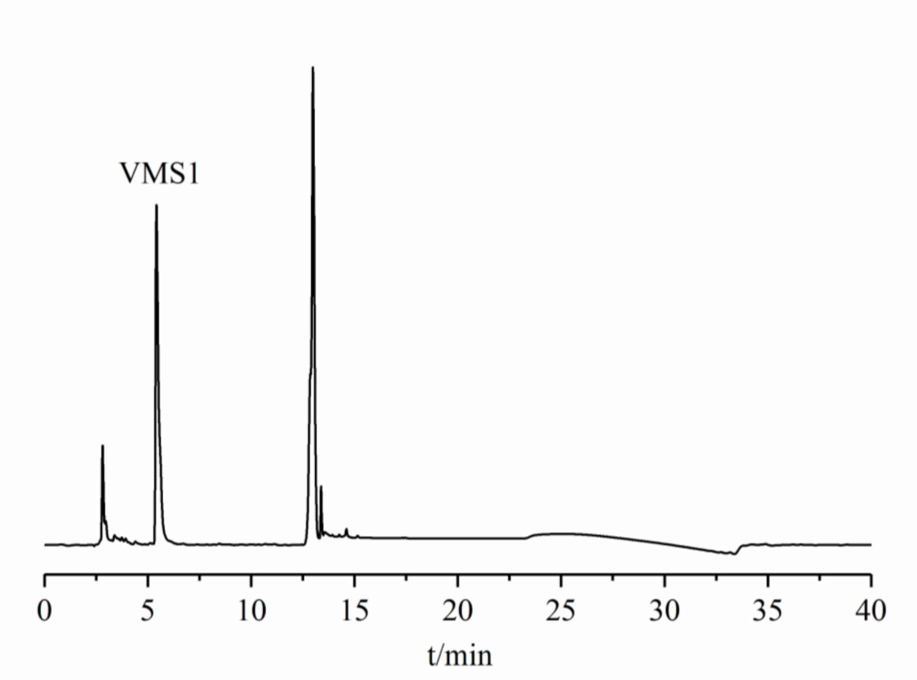

Figure S2. The liquid chromatogram of VMS1.The mobile phase was as follows: A (acetonitrile with 0.1% TFA), B (water with 0.1% TFA). The gradient program was 0 – 9 min, 2% A; 10 – 20 min, 80% A; 30 – 40 min, 2% A. Column temperature was 50℃.

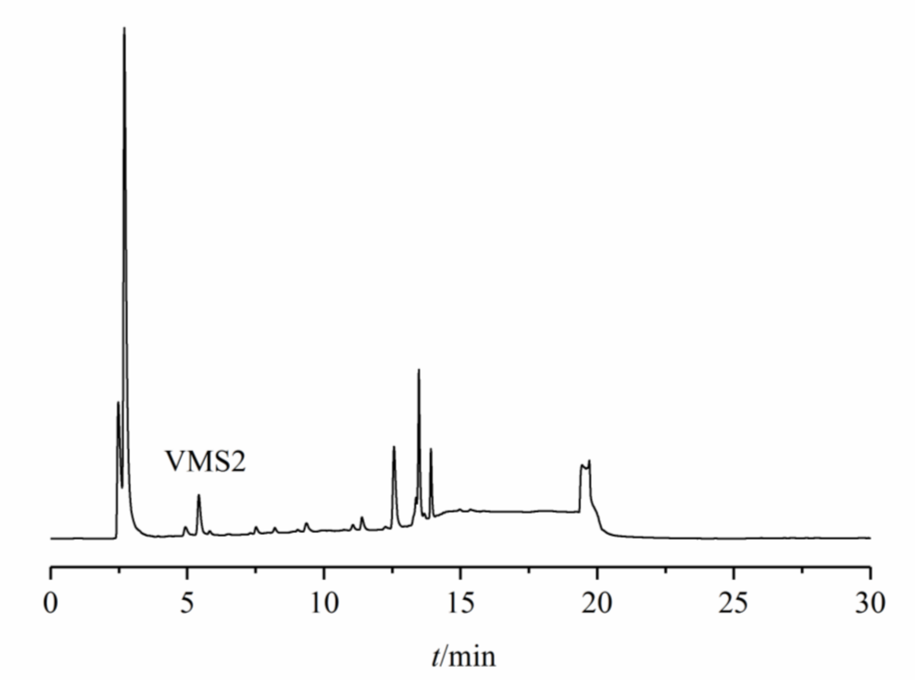

Figure S3. The liquid chromatogram of VMS2. The mobile phase was as follows: A (acetonitrile with 0.1% TFA), B (0.1moL/L Na_2HPO_4). The gradient program was 0 – 10 min, 20% – 40% A; 11–16 min, 80% A; 17 – 22 min, 20% A. Column temperature was 65℃.

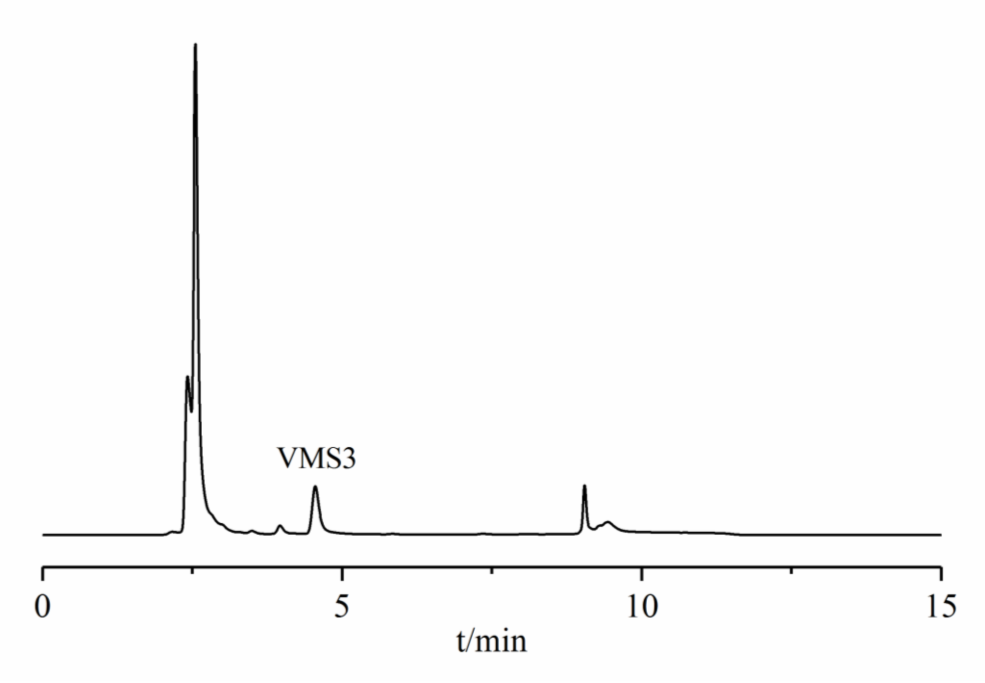

Figure S4. The liquid chromatogram of VMS3. The mobile phase was as follows: A (acetonitrile with 0.05% TFA), B (0.1moL/L Na_2HPO_4, pH = 5, with 0.05% TFA). The gradient program of VMS3 was 0 – 5.5 min, 38% – 43.5% A; 5.6 – 8 min, 50% A; 8.1 – 15 min, 38% A. Column temperature was 50℃.

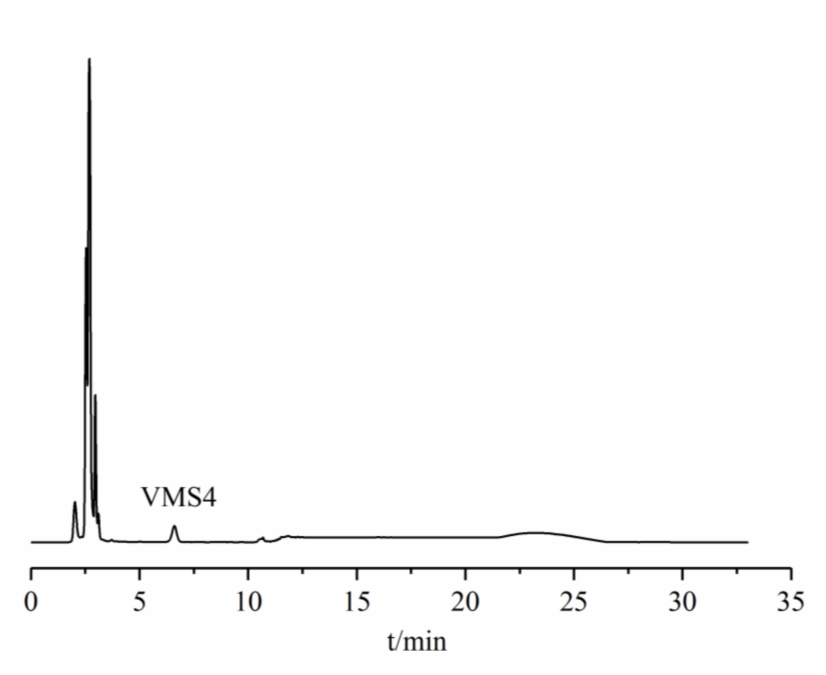

Figure S5. The liquid chromatogram of VMS4. The mobile phase was as follows: A (acetonitrile with 0.1% TFA), B (water with 0.1% TFA). The gradient program of VMS4 was 0 – 7 min, 50% A; 8 – 18 min, 95% A; 23 – 33 min, 50% A. Column temperature was 35℃.

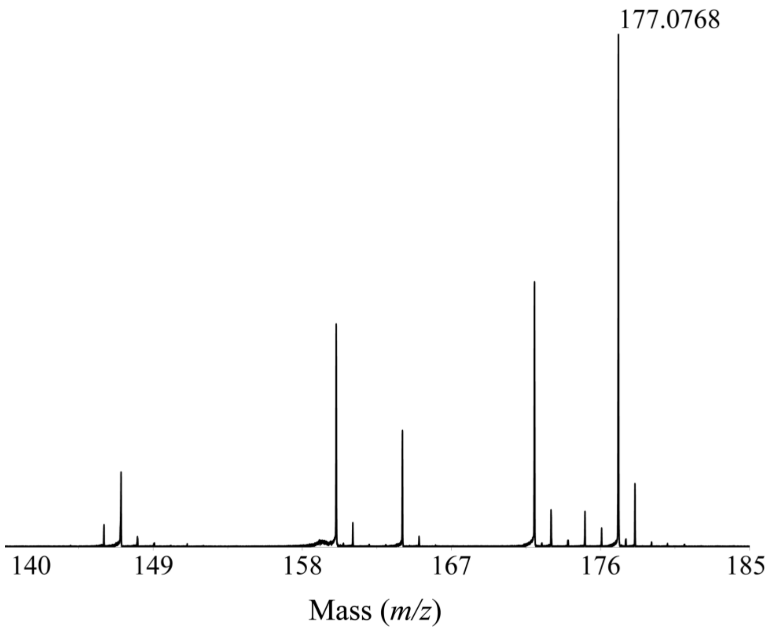

Figure S6. The MALDI-TOF MS spectra of VMS1.

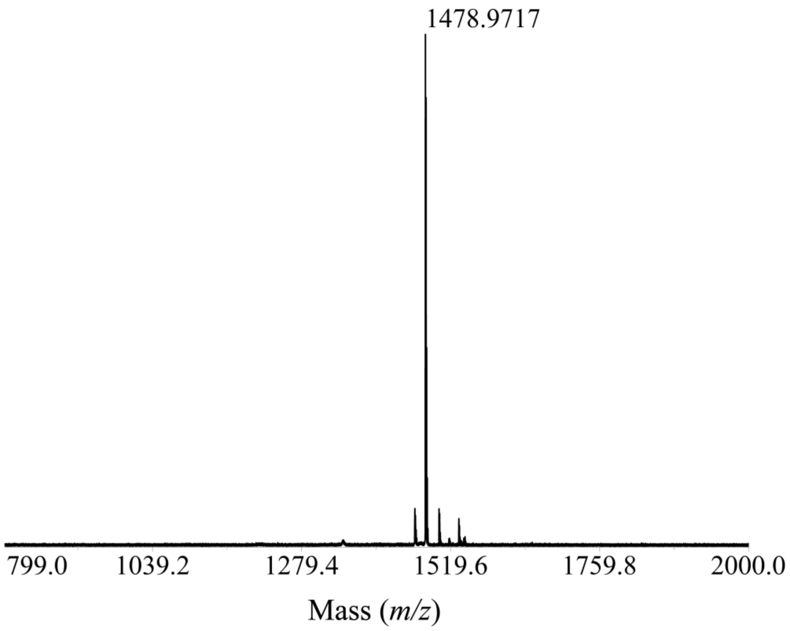

Figure S7. The MALDI-TOF MS spectra of VMS3.

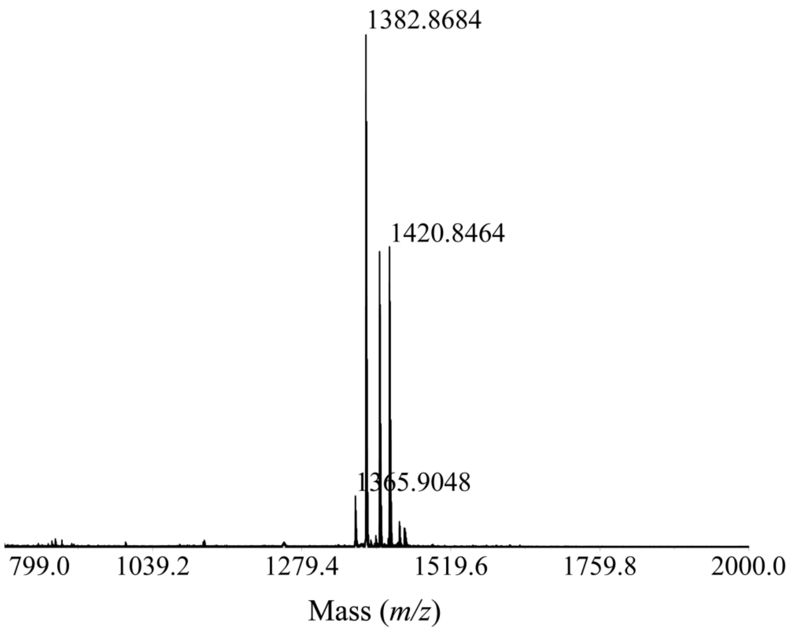

Figure S8. The MALDI-TOF MS spectra of VMS4.
